# ELISPOT Assay for Monitoring Cytotoxic T Lymphocytes (CTL) Activity in Cancer Vaccine Clinical Trials

**DOI:** 10.3390/cells1020111

**Published:** 2012-05-10

**Authors:** Anatoli M. Malyguine, Susan Strobl, Kimberly Dunham, Michael R. Shurin, Thomas J. Sayers

**Affiliations:** 1 Applied and Developmental Research Support Program, SAIC-Frederick, Inc., Frederick National Laboratory for Cancer Research, Frederick, MD 21702, USA; E-Mails: stroblsl@mail.nih.gov (S.S.); dunhamk@mail.nih.gov (K.D.); 2 Departments of Pathology and Immunology, University of Pittsburgh Medical Center, Pittsburgh, PA 15261, USA; E-Mail: shurinmr@upmc.edu; 3 Cancer and Inflammation Program, SAIC-Frederick, Inc., Frederick National Laboratory for Cancer Research, Frederick, MD 21702, USA; E-Mail: sayerst@mail.nih.gov

**Keywords:** ELISPOT, cancer clinical trial, IFN-γ, Granzyme B, Perforin, cytotoxicity, T cell

## Abstract

The profiling and monitoring of immune responses are key elements in the evaluation of the efficacy and development of new biotherapies, and a number of assays have been introduced for analyzing various immune parameters before, during, and after immunotherapy. The choice of immune assays for a given clinical trial depends on the known or suggested immunomodulating mechanisms associated with the tested therapeutic modality. Cell-mediated cytotoxicity represents a key mechanism in the immune response to various pathogens and tumors. Therefore, the selection of monitoring methods for the appropriate assessment of cell-mediated cytotoxicity is thought to be crucial. Assays that can detect both cytotoxic T lymphocytes (CTL) frequency and function, such as the IFN-γ enzyme-linked immunospot assay (ELISPOT) have gained increasing popularity for monitoring clinical trials and in basic research. Results from various clinical trials, including peptide and whole tumor cell vaccination and cytokine treatment, have shown the suitability of the IFN-γ ELISPOT assay for monitoring T cell responses. However, the Granzyme B ELISPOT assay and Perforin ELISPOT assay may represent a more direct analysis of cell-mediated cytotoxicity as compared to the IFN-γ ELISPOT, since Granzyme B and perforin are the key mediators of target cell death via the granule-mediated pathway. In this review we analyze our own data and the data reported by others with regard to the application of various modifications of ELISPOT assays for monitoring CTL activity in clinical vaccine trials.

## 1. Introduction

Monitoring T cell responses in the course of clinical trials is widely used to assess the efficacy of cancer immunotherapy. Selection of an *ex vivo* monitoring technique that provides the best measure of immune reactivity is important in determining potential correlations between clinical and immunologic responsiveness to specific immunotherapy. Standard immunological assays, such as cytokine induction, cell proliferation, and ^51^Cr-release, can detect the overall immune responses in vaccinated patients but are not suitable for efficient evaluation of individual effector cell reactivity. For instance, the tetramer assay identifies the number of epitope-specific cytotoxic T lymphocytes (CTL) [1] but does not necessarily equate to their functional activity [2,3]. To quantitate the functionally active cells, this assay should be combined with intracellular cytokine staining. Assays that can monitor both CTL frequency and function, such as the IFN-γ ELISPOT assay, have gained growing popularity for the immunomonitoring of clinical trials [4,5,6]. However, since Granzyme B and perforin are key mediators of targeted cell death via the granule-mediated pathway [7], the Granzyme B (GrB) and Perforin (Pfp) ELISPOT assays may represent more direct methods for the analysis of cell-mediated cytotoxicity compared to the IFN-γ ELISPOT.

## 2. Mechanisms of Cell-Mediated Cytotoxicity

There is now ample evidence that cytotoxic lymphocytes can be key effector cells involved in the immune-mediated destruction of virally–infected and cancer cells [8]. This cell-contact-dependent cytotoxicity is a hallmark of both cytotoxic T cells (CTL) and NK cells. *In vitro* cytotoxicity assays have shown that 2 major contact-dependent cytotoxic pathways exist: (1) The exocytosis of lytic granules by cytotoxic effector cells, comprising of pore-forming toxin perforin and proapoptotic serine protease granzymes [7], which synergistically kill target cells by activating various lytic pathways; and (2) the production of the TNF family members, such as FasL or TRAIL by the effector cells that induce trimerization or multimerization of their cognate receptors on target cells resulting in their apoptosis [9].

The exocytosis of lytic granules is the major cytolytic pathway employed by both NK cells and CTL [8,9,10]. NK cells are important components of the innate immune response and they constitutively express perforin and granzymes, although expression may be further increased upon stimulation with IL-2. By contrast, CTL are the effector cells of the adaptive immune system. Under normal circumstances, naïve T cells express low or undetectable levels of granule proteins such as perforin or granzymes. However, following specific recognition of antigenic peptides presented on class I MHC molecules (in addition to co-stimulatory and cytokine signaling) by the T cell receptor, both perforin and granzyme levels increase dramatically in T cells over a period of 2–3 days [11,12]. Although a small fraction of CD4+ T cells have been described that express perforin and granzymes [13] these granule proteins are predominantly expressed in highly activated CD8+ T cells. The increase in perforin and granzyme expression upon T cell activation closely parallels with an increase in their cytotoxic potential. After a CTL or NK cell recognizes a target cell, cytotoxic granules move along the microtubules to polarize at the plasma membrane adjacent to the target, where they are secreted into the immunological synapse between the two cells subsequently promoting destruction of the target cell [14].

Both mouse and human NK cells and T cells express a number of different granzymes with unique enzymatic specificities; however granzymes A and B are the most abundant granzymes expressed by cytotoxic lymphocytes [14].

Both granzymes A and B in combination with perforin have been proposed to play a key role in cell-mediated cyotoxicity, however most experimental evidence suggests that granzyme B is the critical granzyme involved in promoting target cell death. Effector cells lacking granzyme B kill targets at a much slower rate than wild-type cells, demonstrating the important role of this protease in executing the timely demise of infected or cancerous cells. The efficiency of granzyme B-dependent killing is largely due to the ability of this protease to activate the target cell’s intrinsic cell death proteases, the caspases, either directly or indirectly [15,16].

In addition to perforin-dependent cyotoxicity, effector lymphocytes can accomplish their cytolytic function against certain target cells *in vitro* by producing pro-apoptotic members of the TNF family of proteins [17]. Binding of these proteins to their receptors on target cells can trigger the apoptotic cascade by activating the extrinsic apoptosis signaling pathway. In order for the target cell to undergo apoptosis in response to TNF family ligands, they must express the appropriate death receptor on their cell surface that, in turn, transmits a strong signal via caspase-8 for subsequent apoptosis to occur. In contrast to granule-mediated cytotoxicity, many cancerous cells are quite resistant to death ligand-induced apoptosis *in vitro*; therefore the effectiveness of this pathway in promoting malignant cell destruction *in vivo* may depend on the biological characteristics of the individual cancer cells.

In many pre-clinical mouse cancer models in which immunotherapy promotes tumor regression, the production of IFN-γ by the host seems to play a crucial role in the ultimate success of the therapy [18]. Interferon gamma is a pleiotropic cytokine with numerous biological effects, however there is little evidence that it can have a direct cytotoxic effect on tumor cells. However, IFN-γ can increase expression of both MHC class I and Fas on the surface of cancer cells, and thus significantly enhancing both granule-mediated and death ligand-mediated cytotoxic effects of CTL [19]. A better understanding of the mechanisms whereby NK cells and CTL promote tumor cell destruction *in vivo* may form a rational basis for additional improving the efficacy of cancer immunotherapy in the future.

## 3. Analyzing Cell-Mediated Cytotoxicity

The most popular assay to date for evaluating cell-mediated cytotoxicity is the chromium release assay (^51^Cr-release). It was first developed in 1968 by Brunner *et al*. [20], and continues to be widely used for testing cellular cytotoxicity. It is based on the passive internalization and binding of the soluble radioactive chromium (^51^Cr) from sodium chromate by target cells in single cell suspensions. Lysis of the target cells by added effector cells results in the release of the radioactive probe into the cell culture supernatant, which can be detected by a gamma counter*.* Though several alternatives have been proposed [21], this assay is considered to be the “gold standard” to measure cell-mediated cytotoxicity. However, while it has benefits of being reproducible and relatively easy to perform, it has several drawbacks: (1) it provides only semi-quantitative data unless it incorporates a limiting dilution component [22]; (2) it has a relatively low level of sensitivity; (3) very often there is a need to stimulate cytotoxic cells several times before testing their lytic activity, and this may distort the composition and activity of the original T cell populations; (4) it does not provide information about the behavior of single cells; (5) there is poor labeling efficacy of some target cell lines; (6) a high spontaneous release from some target cell lines might occur affecting the results; (7) since autologous tumor is difficult to obtain, other surrogate targets must be used, but they may not reflect the actual ability of lymphocytes to lyse autologous tumor cells *in vivo*; (8) its inter-assay variability is considerable; and (9) there are biohazard and disposal problems associated with radioisotope usage. For these reasons, a search for other methods that could replace the ^51^Cr-release has been ongoing.

Several methods have been developed to identify antigen-specific CD8+ T cells. These include, most notably, the use of major histocompatibility complex (MHC) class I tetrameric or pentameric complexes [1,23] intracellular cytokine staining (ICS) [24] and the combination of these methods [25,26]. Both of these techniques can provide valuable information regarding the frequency, phenotype, and/or the functionality of antigen-specific T cells and can be effectively applied to clinical studies. However, these assays do not determine the cytotoxic potentials of the CD8+ T cells since (1) it is well documented that many tetramer-positive cells do not produce detectable levels of IFN-γ after direct ex vivo stimulation with cognate peptide; (2) CD8+ T cells that produce cytokine after stimulation are not always cytotoxic; and (3) tetramer-positive cells occasionally fail to kill targets expressing the specific antigenic peptide epitope [27,28]; (4) The specific cytokines may be detected by ICS, but they will not produce any biological effects until secreted. Furthermore, cytotoxic lymphocytes mediate cell death by Fas-FasL interaction and by the secretion of cytotoxic molecules including GrB and Pfp and this function of CTL and NK cells should be appropriately assessed in the immunomonitoring protocols.

## 4. ELISPOT Assays

Assays that can monitor both CTL frequency and function, such as the IFN-γ enzyme-linked immunospot assay, are commonly used in basic research and for monitoring cancer clinical trials [4,5,6]. The ELISPOT assay was introduced in 1983 by Sedgwick and Holt [28] and since that time more than 3,000 papers, in which an ELISPOT assay was used, were published. The ELISPOT assays enumerate antigen-specific lymphocyte frequency by measuring secretion of specific proteins that are the critical components of the specific pathways utilized to mediate lysis of target cells. ELISPOT assays detect locally secreted cytokine molecules by means of antibody-coated plastic plates or membranes that capture the secreted cytokine derived from the productive interaction of the effector cell and its target cell. There are numerous advantages to utilizing the ELISPOT assays over the standard ^51^Cr -release assay, including: (1) the ELISPOT assays enumerate antigen-specific lymphocyte frequency, as well as function, by measuring secretion of a specific protein and, as such, the ELISPOT assays are both qualitative and quantitative; (2) they detect functionally relevant molecules upon the specific stimulation of effector cells; (3) the ELISPOT assays use low numbers of effector cells to accurately assess their activity, which is quite beneficial for monitoring clinical trials with limited numbers of patients’ cells available; (4) the high sensitivity and specificity of the ELISPOT assays are also beneficial for clinical monitoring; (5) additionally, the problems associated with the labeling efficiency of targets are not a concern for the ELISPOT assays. These and other benefits of ELISPOT technique have been reviewed recently by Lehmann and Zhang [29], who also concluded that the assay is an excellent choice for performing clinical monitoring.

## 5. IFN-γ ELISPOT Assay

It has previously been shown that the results of IFN-γ ELISPOT often correlate well with the results of ^51^Cr-release assay [30] and a variant of the LDA, the multiple microculture assay [31]. For several years the IFN-γ ELISPOT assay has been widely used for monitoring multiple cancer vaccine clinical trials, including peptide and whole tumor cell vaccination and cytokine treatment protocols [32,33,34]. However, using the IFN-γ ELISPOT assay alone may not be sufficient since certain non-cytotoxic cells can secrete IFN-γ, whereas CTL with proven lytic activity do not always secrete IFN-γ [29]. This might explain, at least in part, why, based on large amounts of clinical data, though appreciable numbers of treated patients exhibited tumor-specific T cell responses, only a small percentage of these patients experienced clinically noticeable tumor regression. For instance, our data using IFN-γ ELISPOT assay revealed that 75% of melanoma patients vaccinated with antigenic peptide demonstrated specific immune responses [35]. However, according to the analysis of more than 1000 vaccine treatments conducted in the Surgery Branch of NCI and several non-NCI trials, only 3.3% of patients demonstrated objective clinical responses based on standard oncologic reporting criteria [36]. There was no difference in the levels of anti-tumor antigen-specific T cells evaluated in IFN-γ ELISPOT assay in vaccinated melanoma patients who recurred compared with those who remained disease-free [37]. Furthermore, in a recent clinical trial with vaccinated glioma patients, 74% of patients demonstrated specific responses against targeted glioma-associated antigens. However, only two of 19 patients (9%) had objective clinical responses, and both were classified as non-responders in the ELISPOT assay [38]. Thus, there is often a poor correlation between the IFN-γ ELISPOT assay and clinically relevant immune responses and better and more reliable assays are urgently needed.

## 6. Granzyme B ELISPOT Assay

The Granzyme B ELISPOT assays may provide a more direct measure of cell-mediated cytotoxicity compared to the IFN-γ ELISPOT since GrB is one of the key mediators of target cell death induced by the granule-mediated lytic pathways. Thus, the release of GrB by cytolytic lymphocytes upon effector-target interactions may be a more specific indicator of CTL and NK cytotoxic ability than IFN-γ secretion.

The GrB ELISPOT assay was initially developed by Rininsland *et al.* [39]. The authors used GrB transfected Chinese hamster ovary (CHO) cells and T cell lines and demonstrated that only activated T cells secrete GrB within 4 h after antigen stimulation. Side-by-side comparison showed that the GrB ELISPOT assays had higher sensitivity than the ^51^Cr release assay and also measured the frequencies of GrB-secreting cells directly. When freshly isolated PBMC from HIV patients were tested by GrB ELISPOT, a specific reactivity to HIV peptides was also demonstrated.

In order to apply the GrB ELISPOT assay for monitoring cancer vaccine trials, our laboratory has optimized the assay for various cytolytic cells [40]. These cells included established allogeneic CTL cell lines (αEN-EBV and αJY) and short-term (one week) induced anti-peptide CTL. The assay measured stimulated GrB secretion when CTL were incubated with their specific targets. We investigated if evaluating GrB secretion in the ELISPOT assay can accurately assess the frequency of CTL. We addressed this issue by performing serial dilutions of the effector cells. The various dilutions of αEN-EBV CTL were then run against a constant number of specific and non-specific target cells in the GrB ELISPOT assay. A significant correlation between the number of plated CTL and the number of spots per well was observed, thereby suggesting that the GrB ELISPOT assay can accurately enumerate the precursor frequency of GrB-secreting cells. Similar results were observed with αJY CTL. Our findings were in excellent agreement with those in the report that utilized GrB transfected CHO cells and T cell lines as effector cells [41]. To study the specificity of GrB secretion by αFMP-CTL, we tested CTL reactivity against FMP-pulsed C1R.A2 (specific targets), as well as non-pulsed and MART-1 pulsed C1R.A2 cells (non-specific targets) in the GrB ELISPOT assay. K562 were utilized as a control for NK activity. Granzyme B secretion was antigen-specific, as only wells in which CTL were incubated with FMP-pulsed CIR.A2 cells contained a substantial number of spots. To further confirm that we were measuring CTL activity, we removed CD8+ cells from the cultures using anti-CD8 mAb and magnetic beads and abrogation of both GrB and IFN-γ secretion was observed.

Since cell-mediated cytotoxicity has conventionally been measured using the standard ^51^Cr-release assay, we compared the GrB ELISPOT assay to the ^51^Cr -release and IFN-γ ELISPOT assays to evaluate its ability to quantify CTL lytic responses. Both ELISPOT assays were significantly more sensitive than the ^51^Cr -release assay. When the optimal number of CTL are used in each individual assay, the amount of GrB and IFN-γ secreting cells in the ELISPOT assays and cytotoxicity in the ^51^Cr -release assay have shown excellent cross-correlation (R2 = 0.95).

Interestingly, the dynamics of GrB and IFN-γ secretion differ. Granzyme B secretion was detectible as early as 10 min after initial contact of effectors and targets. Significant numbers of GrB spots were observed within 30 min of incubation with maximal secretion at 4 h. Longer incubation times did not increase GrB secretion. In contrast, measurable amounts of IFN-γ spots were observed only after 1 h of incubation, with significant IFN-γ secretion measured at 4 h. Similar dynamics were observed for the ^51^Cr-release assay. When we applied GrB ELISPOT assay for the evaluation of NK activity, practically the same dynamics was shown for both ELISPOT assays and for the ^51^Cr-release assay [42].

The difference in the observed pattern of GrB and IFN-γ secretion parallels the well defined dynamics of CTL effector functions. When CTL or NK cells interact with their target cells, IFN-γ secretion results from de novo protein synthesis upon activation followed by secretion within hours, whereas GrB is released very rapidly (within minutes) from preformed granules. Using NK cells, we have shown that treatment with Brefeldin A, a protein secretion blocker, significantly decreases IFN-γ but not GrB production. In contrast, BAPTA-AM, which sequesters intra-cellular calcium and therefore inhibits degranulation, only abrogated GrB secretion [42]. The fact that the number of effectors spontaneously secreting GrB is somewhat higher than the number secreting IFN-γ suggests that the GrB measured in the ELISPOT could be present in preformed granules. Granzyme B is consistently expressed in activated cytolytic cells, especially CD8+ CTL. In activated CTL, cross-linking of the TCR is sufficient to trigger the release of GrB from preformed granules, as well as promoting the rapid synthesis of new GrB protein.

Recently, a flow cytometric assay based on CD107a (lysosomal-associated membrane protein-1) mobilization was developed to measure degranulation of cytolytic cells [43]. CD107a is a vesicle membrane protein of cytolytic granules that is transiently expressed on the surface of effector cells during degranulation. Correlations between direct lytic ability and surface expression of CD107a on effector cells have been shown, indicating that CD107a expression is a reliable measure of cytolytic capacity. CD107a expression strongly correlated with GrB secretion confirming that the GrB ELISPOT assay is an excellent measure of cytotoxic capacity mediated by effector cell degranulation [44,45].

Both the GrB and IFN-γ ELISPOT assays are superior alternatives to the ^51^Cr-release assay to test CTL response. However, when compared with the IFN-γ ELISPOT, the GrB ELISPOT assay is more rapid and may be a more direct measure of antigen specific CTL lytic activity. Our studies with samples from melanoma patients vaccinated with gp100:209M peptide suggest that the GrB ELISPOT assay may be successfully applied to evaluate CTL precursor frequency, and reactivity in the GrB ELISPOT was more closely associated with cytotoxicity as determined by the ^51^Cr -release assay than either the tetramer or IFN-γ ELISPOT assays [35].

The presence of antigen-specific T cells that can recognize tumor cells is not always sufficient to mediate anti-tumor responses. The clinical efficacy of cancer vaccines likely depends on several factors including the specificity, functional quality, and the magnitude of the induced anti-tumor T cell response. Assays that assess the function as well as the frequency of tumor-reactive T cells are crucial for evaluating and further developing cancer vaccine therapies. We have investigated whether the GrB ELISPOT assay can be applied to monitor the frequency and activity of CTL in peripheral blood mononuclear cells (PBMC) from patients with cancer. PBMC from melanoma patients vaccinated with an HLA-A2*0201 binding peptide from the gp100 protein (gp100:209-2M; this peptide contains the same TCR contact residues as gp100:209, but is modified to have a higher affinity binding anchor for HLA-A*0201) were utilized [35]. We compared peptide-stimulated reactivity of PBMC from vaccinated cancer patients in the GrB and IFN-γ ELISPOT assays, as well as in the tetramer and ^51^Cr-release assays. Four distinct response patterns were observed among the patients that were tested in all four assays. Five of the 16 patients were unresponsive in all four assays. Eleven responsive patients could be further categorized. Four were positive in all four assays, three were positive in the tetramer, IFN-γand GrB assays, and four were positive in only the tetramer and the IFN-γassays. These data patterns demonstrate that vaccination can elicit differences in immune responses among individual patients. When patients were tested for immune responsiveness at different time points after vaccination, very limited GrB secretion was observed in response to targets pulsed with the control peptide (g280) regardless of time after vaccination. These data demonstrate the specificity of the GrB ELISPOT, and its ability to measure immune responses elicited after vaccination.

Correlations between the tetramer, IFN-γ and GrB ELISPOTs and the ^51^Cr-release assays have been shown to be different. The Phi Coefficient demonstrated that the IFN-γ and tetramer assays perfectly correlated. The GrB ELISPOT assay was significantly associated with all three of the other assays. However, the ^51^Cr-release assay only significantly correlated with the GrB ELISPOT. These data suggest that the GrB ELISPOT is a viable alternative to the ^51^Cr-release assay and may measure different effector cells than do the tetramer and IFN-γ assays. Some patients demonstrated GrB secretion in the absence of significant cytotoxicity measured via the ^51^Cr-release assay demonstrating that the GrB ELISPOT assay may be more sensitive.

Additionally, differences were observed in the ability of patients’ PBMC ability to secrete IFN-γ as compared to GrB when they were tested against g209 and higher affinity g209-2M peptides. Limited differences between the amount and the time course of IFN-γ secretion were observed with respect to stimulation with g209 *versus* g209-2M. By contrast, patients’ PBMC secreted greater amounts of GrB in response to the higher affinity vaccinating peptide, g209-2M. Our data suggest that peptide affinity may modulate the type of immune response mediated by antigen-specific CTL, and that higher affinity peptides are more efficient at stimulating cytotoxic effector functions. In support of this hypothesis, Akiyama *et al*. [46] demonstrated that only the higher affinity CMV peptides could elicit potent human CTL. Wong *et al*. [47] suggest that the signaling threshold for IFN-γ secretion may be lower than the signal needed to initiate lytic ability. Additionally, the affinity of peptides binding to MHC has been shown to regulate the phenotype of murine CTL [48].

Although a limited number of patients were analyzed and direct comparisons were not made to clinical outcomes, one can speculate that vaccination is inducing the generation of different populations of effector cells or that the same effector cells are undergoing alternative differentiation over time due to vaccination. Preliminary findings utilizing *in vitro* stimulated CTL in a simultaneous GrB and IFN-γ dual color ELISPOT approach show distinct populations of IFN-γ only, GrB only and dual secreting cells in response to relevant peptide stimulation [our unpublished data]. We also enumerated more antigen-specific T cells in the tetramer assay than either of the ELISPOT assays. Overall, this suggests that some of the antigen-specific cells detected in our assays may be persisting in the patients in a nonfunctional state. In support of this hypothesis, antigen-specific T cells in viral and melanoma models quantified by tetramer staining have a diminished capacity to secrete IFN-γ or mediate cell-induced cytotoxicity and quiescent tumor-specific CD8^+^ T cells have been found in the circulation of immunized melanoma patients [49,50]. Therefore, it is important to complement the tetramer assay with assays that measure antigen-specific T cell functional ability. Significantly more antigen-specific T cells produced IFN-γ than GrB suggesting that in addition to the magnitude of the immune response, the type of immune response generated to peptide vaccination may also be important.

Granzyme B ELISPOT assay was applied to monitoring immune responses in cancer patients in several publications (Table 1).

**Table 1:** Application of GrB ELISPOT assay for monitoring antitumor response in cancer patients.

Cancer/number of patient tested	Antigen	Patient vaccination	Ex vivo stimulation/effector cells	Results of GrB ELISPOT	Ref
Breast cancer/3	Bcl-x_L_	no	One round/PBMC	CTL responses against Bcl-x_L173–182_ in 2/3 patients, correlated with IFN-γ ELISPOT and ^51^Cr release assay	[51]
Breast cancer /7	no	no	Expansion of γδ T cells	Average release GrB by γδ T cells significantly higher in normal donors, as well as IFN-γ and ^51^Cr release	[52]
Colon cancer/5	CP1 (Cancer-placenta)	no	Two rounds in ex vivo stimulation/isolated CD8+T cells	CD8+ response in 3/5 tested patients; correlated with IFN-γ ELISPOT	[53]
Head and neck squamous cell carcinoma/8	TAA RHAMM and G250	no	MLC of isolated CD8+ T cells and APC	Anti-RHAMM CD8+ T cells response against tumor cells in 4/5 patients and anti-G250 CD8+ cells in 3/4. No correlation with IFN-γ ELISPOT	[54]
Hepatocellular carcinoma/5	NY-ESO -1b	no	2–3 rounds activation of isolated CD8+ T cells	Specific CD8+ T response in 2/5 patients; correlated with IFN-γ ELISPOT	[55]
Malignant melanoma/16	Gp100	Gp100 peptide vaccine	No/PBMC	7/16 specific response *vs.* 11/16 for tetramer and IFN-γ ELISPOT and 4/16 ^51^Cr release. Correlation with vaccination course	[35].
Malignant melanoma/7 *	Gp100	Gp100 peptide vaccine	PBMC pre selected for positive response with or without one round ex vivo activation	Correlation with IFN-γ ELISPOT, ^51^Cr release	[44]
				and CD107a/Annexin V flow cytometric assay	
Chronic lymphocytic leukemia/5	RHAMM derived epitope R3	R3 peptide vaccination	No/PBMC	In response to vaccination 4/5 of tetramer-positive samples produced both GrB and IFN-γ	[56]
Acute myeloid leukemia/3	PRAME derived peptide	DC pulsed with peptide	MLC of isolated CD8+ T cells	1/3 response to vaccination	[57]
Pancreatic cancer/7	MUC1	DC pulsed with peptide	PBMC	2/7 response to vaccination; correlated with IFN-γ ELISPOT	[58]
Malignant melanoma/1	T-helper epitope of MART-1	T-helper epitope of MART-1	Isolated CD4+ T from PBMC activated with peptide-pulsed DC	Response to vaccination	[47]
Chronic myeloid leukemia/9	RHAMM derived epitope R3	Allogeneic cell transplantation	Isolated CD8+ T cells	Response to R3 in 67% (6/9) of the CML patients after allo-SCT and 24% (8/34) of healthy donors	[59]
Healthy donors/34					

* Seven samples were from the patients with positive immune response from previous study (see the row above).

All together, including our studies, 69 patients were tested, from which 41 were from cancer vaccine clinical trials. 21 of these patients demonstrated increase of GrB production in ELISPOT assay after vaccination; in most cases it correlated with IFN-γ and ^51^Cr-release. Application of GrB ELISPOT for monitoring AIDS patients has been reviewed by Lehmann and Zhang [29].

## 7. Perforin ELISPOT Assay

Since the role of perforin (pfp) in immuno-surveillance and rejection of tumors has been well established, there is great interest in using Pfp ELISPOT for clinical monitoring. The Pfp ELISPOT assay was established by Zuber *et al.* [60], who produced anti-pfp mAb and developed the Pfp ELISA and ELISPOT assays. Three mAbs were generated and shown to react with unique determinants of pfp and recognized intracellular pfp in human PBMC. Pfp ELISA and ELISPOT assays were developed utilizing two of the mAbs for capture and the third mAb for detection. The ELISPOT assay displayed greater detection sensitivity than the ELISA when the YT cell line was used as effector cells. Pfp release by CTL clones in the ELISPOT correlated with the ^51^Cr-release cytotoxicity assay. Pfp ELISPOT and ELISA were used to quantify CTL response to common viruses as well.

Data on the application of Pfp ELISPOT to the monitoring of cancer patients is limited. Andersen *et al.* [61] studied spontaneous immune responses in HLA-A24+ patients with various cancers to HLA-A24-restricted survivin epitopes. Isolated CD8+ T cells were tested both in IFN-γ and Pfp ELISPOT assays and positive responses were detected in both assays in 6 of 8 tested patients. Recently, Vetsika *et al.* [62] investigated the specific T cell immune response against Vx-001, an HLA-A*0201 restricted telomerase (TERT)-specific anti-tumor vaccine, in HLA-A*0201-expressing patients with various types of advanced solid tumors. A specific immune response was evaluated by IFN-γ and perforin ELISPOT and intracellular cytokine staining assays. From 6 patients with positive IFN-γ ELISPOT results, 5 also had a positive response in the pfp ELISPOT assay.

The clinical data presented show that GrB and Pfp ELISPOT assays are applicable for monitoring cancer vaccine trials. In most cases the assays correlated with the IFN-γ ELISPOT assay, which is widely used for clinical monitoring. Only one publication showed that the reactivity in the GrB ELISPOT was more closely associated with cytotoxicity in the ^51^Cr-release assay than the tetramer or IFN-γ ELISPOT assays. Moreover, the higher affinity g209-2M peptide elicited greater GrB secretion than the native g209 peptide, while this difference was not observed with IFN-γ secretion [35]. These results show that simultaneous use of the GrB ELISPOT assay with other immunological assays may provide important additional immunological insights into the patient’s responses to cancer vaccines*.* Unfortunately, to date a limited number of cancer patients have been monitored by IFN-γ, GrB and/or Pfp ELISPOT assays run in parallel. This does not allow us to make final conclusion regarding the relative value of these assays. However, taking into account that GrB and Pfp ELISPOTs directly measure cytotoxic activity of NK and CTL, and since Granzyme B and perforin are key mediators of target cell death via the granule-mediated pathway, we believe that these assays maybe valuable for monitoring cancer vaccine trials in the future.

## Conclusions

The therapeutic use of the immune system to attack tumor cells has been a longstanding vision among tumor immunologists and clinicians. However, the evaluation and comparison of immunotherapeutic clinical trials requires a thorough and detailed analysis of immune responsiveness in treated patients. Unfortunately to date, the appropriate immunologic responses induced by vaccination that result in effective tumor rejection have not been elucidated. One significant unresolved issue in modern immunotherapy is the observation that the tumor-specific cellular immune response that often follows a course of immunotherapy does not always lead to clinically proven cancer regression. This despite the ability of patients’ T cells to produce immunostimulatory cytokines and generate tumor-specific cytotoxic lymphocytes able to recognize and efficiently kill tumor cells *ex vivo*. Based on large amounts of clinical data, appreciable numbers of treated patients exhibited the tumor-specific T cell response, while only a small percentage of these patients experienced clinically noticeable tumor regression [63]. This disappointing lack of a correlation between the tumor-specific cytotoxic immune responses and the clinical efficacy of immunotherapy may be explained in part by the lack of accepted uniform standards for immunological monitoring of clinical trials. Also, it is very unlikely that the analysis of any single immunological parameter is sufficient to provide clinically feasible information about the extremely complex interactions between different effector and tumor cells. It is conceivable that the combination of assays, including various modifications of ELISPOT assays, which allows measurement of several immunological parameters, may be advantageous for the monitoring of patients in clinical trials.
